# Retinoid X Receptor Antagonists

**DOI:** 10.3390/ijms19082354

**Published:** 2018-08-10

**Authors:** Masaki Watanabe, Hiroki Kakuta

**Affiliations:** Division of Pharmaceutical Sciences, Okayama University Graduate School of Medicine, Dentistry and Pharmaceutical Sciences, 1-1-1, Tsushima-naka, Kita-ku, Okayama 700-8530, Japan; ph424140@s.okayama-u.ac.jp

**Keywords:** Retinoid X receptor, RXR, ligands, modulators, antagonists, structural classification, heterodimers, non-permissive, permissive, tRXR

## Abstract

Retinoid X receptor (RXR) antagonists are not only useful as chemical tools for biological research, but are also candidate drugs for the treatment of various diseases, including diabetes and allergies, although no RXR antagonist has yet been approved for clinical use. In this review, we present a brief overview of RXR structure, function, and target genes, and describe currently available RXR antagonists, their structural classification, and their evaluation, focusing on the latest research.

## 1. Introduction

**Figure 1:**
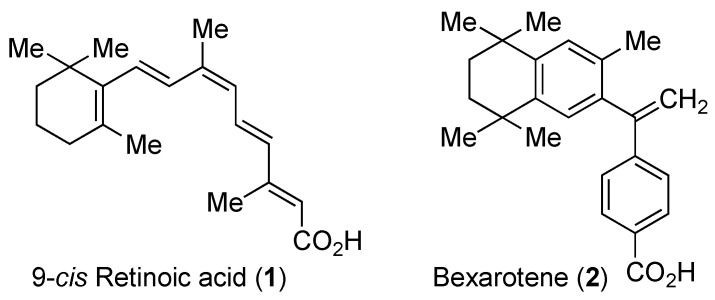
Chemical structures of 9-*cis*-retinoic acid (**1**) and bexarotene (**2**).

Retinoid X receptors (RXRs; NR2B1–3) are nuclear receptors that function either as homodimers or as heterodimers with other receptors such as peroxisome proliferator-activated receptors (PPARs; NR1C1–3), liver X receptors (LXRs; NR1H2–3), or farnesoid X receptor (FXR; NR1H4), and others [1,2,3]. RXR heterodimers that can be activated by RXR agonists alone are known as permissive heterodimers [4]. 9-*cis*-Retinoic acid (**1**, Figure 1) is a potent natural agonist toward RXRs, but also works as an activator of retinoic acid receptors (RARs) [5]. The RXR synthetic agonist bexarotene (LGD1069, Targretin^®^, **2**, Figure 1) is used for the treatment of cutaneous T cell lymphoma (CTCL) [6], but, on the other hand, no RXR antagonist has yet entered clinical use, even though anti-type 2 diabetes [7] and anti-allergy activities [8] have been found in animal models. At present, RXR antagonists are mainly employed as analytical tools in studies of RXR function. In this review, we will first present a brief overview of the RXR structure, function, and target genes, and then describe currently available RXR antagonists, their structural classification, and their evaluation, focusing on the latest research.

## 2. The RXRs

Among the 48 members of the nuclear receptor superfamily that have identified by sequence alignment and phylogenetic tree construction [3,9], 24 are ligand-binding receptors. These include three different subtypes of RXR, i.e., RXRα, RXRβ, and RXRγ, which are encoded by distinct genes [3] (Table 1). Historically, these receptors have been named after their ligands. However, the Nuclear Receptor Nomenclature Committee has recommended a systematic nomenclature based on genome analysis [3]. Thus, RXRα is designated as NR2B1, RXRβ as NR2B2, and RXRγ as NR2B3; in this review, we retain the established nomenclature. Each RXR has two isoforms: RXRα1/α2, RXRβ1/β2, and RXRγ1/γ2 [10]. RXRα exists in liver, lung, muscle, kidney, epidermis (major subtype), and intestine, while RXRβ is distributed ubiquitously. On the other hand, RXRγ1 is expressed in the brain and muscle, while RXRγ2 is highly expressed in cardiac and skeletal muscles [10]. Most research has so far been focused on RXRα; one reason for this may be that the functions of the RXR subtypes are the same, even though their distributions are different. RXRα was the first RXR subtype to have its structure determined by X-ray crystallography [11].

**Figure 2:**
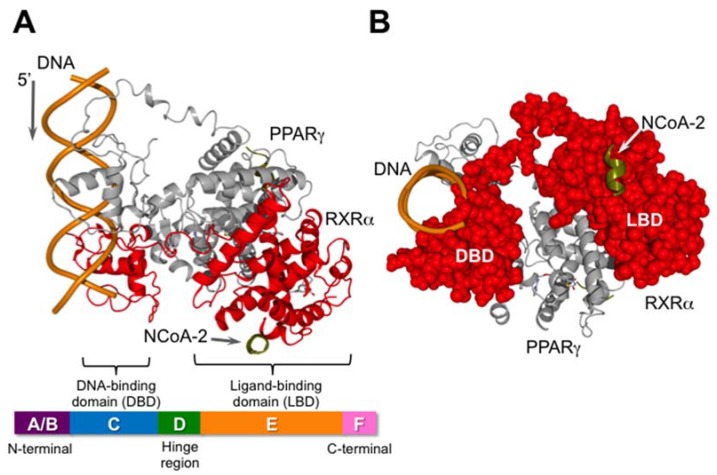
Schematic illustration of the nuclear receptor heterodimer PPARγ/RXRα and its co-activator (NCoA-2: nuclear receptor co-activator 2) bound to DNA. This figure was created using PDB coordinates from [13] (pdb: 3DZY). RXR is shown in red. The X-ray data was obtained using only a part of N-CoA2, consisting of EKHKILHRLLQDSY. (**A**) View from the side. The bar at the bottom is a schematic illustration of the general domain structure of nuclear receptors; (**B**) View from the 3′-end of the DNA. RXR is shown as a CPK model in red.

RXRs, like other nuclear receptors, consist of six domains A, B, C, D, E, and F (Figure 2) [3]. The N-terminal A/B region has a transcriptional activation function and is referred as AF-1 (Figure 2, Table 1). AF-1 can operate in a ligand-independent/dependent manner; i.e., it is controlled by ligand binding to the ligand-binding domain (LBD) (Figure 2, Table 1) in the full-length receptor, but when located outside of the receptor, it acts in a ligand-independent manner [3]. Next, the C domain acts as a DNA-binding domain (DBD) (Figure 2, Table 1), which contributes to the response element specificity for recognition of the target gene. Dimerization of RXR with itself or a heterodimer partner is caused by strong interactions between the LBDs of the interacting partners, as well as binding of the two DBDs. RXR homodimers bind to RXR response elements (RXREs) composed of a direct repeat of hexad half-sites (A/G)G(G/T)TCA separated by one nucleotide as a spacer (DR-1 element, direct repeat with 1 nucleotide) (Table 2). RXR heterodimers also bind preferentially to specific hormone response elements (HREs), which are composed of two hexad half-sites arranged as tandem repeats. The specificity of each dimer for the target DNA is based not only on the DNA sequences of the two half-sites, but also on the geometry, spacing, and relative orientation of the half-sites in the HRE [12,13,14,15]. Other response elements with different numbers of spacer nucleotides, DR2, DR3, DR4, DR5, and others, also exist. The RXR LBD can adopt multiple conformations, providing the dimerization domain with sufficient flexibility to occupy the partner receptor [14,15]. Interestingly, RXRs can form RXR tetramers with high affinity at protein concentrations higher than about 70 nM [16]. Noy and collaborators presented evidence that binding of the apo-RXR homotetramer to two RXREs, which were separated by 250 base-pairs in a 382 base-pair sequence, permitted transcriptional regulation by DNA-looping [17]. The reason why one RXR homotetramer can bind to two different RXREs is that their DBDs are exposed. Indeed, the inhibition of mammary carcinoma cell growth by RXR ligands stems from the ability of these compounds to regulate the oligomeric state of RXR, and is independent of the direct intrinsic transcriptional activity of the receptor [18]. Compounds that trigger dissociation of RXR tetramers may comprise a novel class of anti-carcinogenic agents.

Target genes of RXR heterodimers are dependent on the identity of the heterodimer partner. On the other hand, in the case of RXR homodimers, the DBDs bind to natural DR1 elements for the calcitonin receptor activity-modifying protein 2 (*Ramp2*), the NR subfamily 1, group D, member 1 (*Nr1d1*) and the glycerophospho-diester phosphodiesterase 1 (*Gde1*) genes, as well as the malic enzyme PPRE gene (*MEp*) [15,19,20]. Since both RXR and PPAR bind to DR1, RXR homodimers can bind not only RXRE, but also PPRE [20].

Domain D acts as the binder and cushion of domains C and E. Domains E/F are referred as the ligand-binding domain (LBD) (Table 1, Figure 2). The LBD contains four structurally distinct, but functionally linked surfaces: (1) a dimerization surface with a partner; (2) the ligand-binding pocket (LBP) for lipophilic small molecules; (3) a co-regulator binding surface; and (4) a ligand-dependent activation function helix 12 (termed AF-2) (Figure 2, Table 1) [3]. Activation of RXRs occurs when an agonist binds to the LBP and induces a conformational change in the LBD [21]. The resulting conformation allows recruitment of co-regulatory complexes, which contain chromatin-modifying enzymes required for transcription, RNA polymerase II, and general transcription factors [22,23]. RXR heterodimers with PPARs, LXRs, or FXR, which can be activated by RXR agonists alone, are known as permissive heterodimers [4]. In contrast, RXR heterodimers with RAR or TRs cannot be activated by RXR agonists alone, and are termed non-permissive. The difference between permissive and non-permissive heterodimers arises from the strong constitutive interaction between the unliganded non-permissive hetero partner and co-repressors [24]. Unliganded permissive heterodimer partners, such as PPAR or LXR, do not have a strong constitutive interaction with co-repressors [25], so their RXR heterodimers can be activated by an RXR agonist alone.

Small molecules or compounds that bind reversibly to nuclear receptors into the C-terminal ligand-binding pocket (LBP) are defined as “nuclear receptor ligands” [3]. Due to the ability to alter activity of the receptors, these are often termed “receptor modulators” [26]. However, since there are small molecules that bind to a different site from the LBP, the definition of “nuclear receptor modulators” should be broadened as compounds that bind to nuclear receptors (Table 1). Nuclear receptor ligands are classified into three categories; agonists, inverse agonists, and antagonists (Table 1). Agonists are defined as compounds that bind to the LBP and activate the receptor. Inverse agonists are compounds that bind to the LBP and result in a conformational change that reduces the basal level of activity (reduces basal co-activator binding). In contrast, Antagonists simply bind to the LBD and prevents the conformational change that an agonist would cause, thus preventing co-activator recruitment and subsequent stimulation of transcription. The definition of other terms is listed in Table 1.

RXR antagonists interfere with the binding of RXR agonists to RXRs. Although some subtype-preferential agonists and antagonists have been reported [27,28,29], their selectivities are not sufficient to allow their use as pharmacological tools [30]. The main reason for the difficulty in developing highly selective RXR ligands may be that the amino acid residues of helices (H) 3, 5, 7, and 11, and the β-turn, which form the ligand-binding pocket, are highly conserved in RXRα, β, and γ.

## 3. Representative RXR Antagonists

RXR antagonists are classified into three categories; (1) compounds having a long-chain alkoxy group introduced to an RXR agonist structure as a scaffold (Table 3); (2) compounds possessing another side-chain group instead of the alkoxy group introduced to an RXR agonist structure as a scaffold (Table 4); and (3) compounds discovered from among natural products or by docking simulation or high-throughput screening (Table 5). The common structure of RXR agonists is composed of three parts: a hydrophobic moiety composed of a tetramethyltetraline structure, an acidic moiety composed of trienoic acid, benzoic acid, nicotinic acid, or pyrimidinecarboxylic acid, and a linking moiety between the two.

### 3.1. RXR Antagonists Having a Long-Chain Alkoxy Group

**Figure 3:**
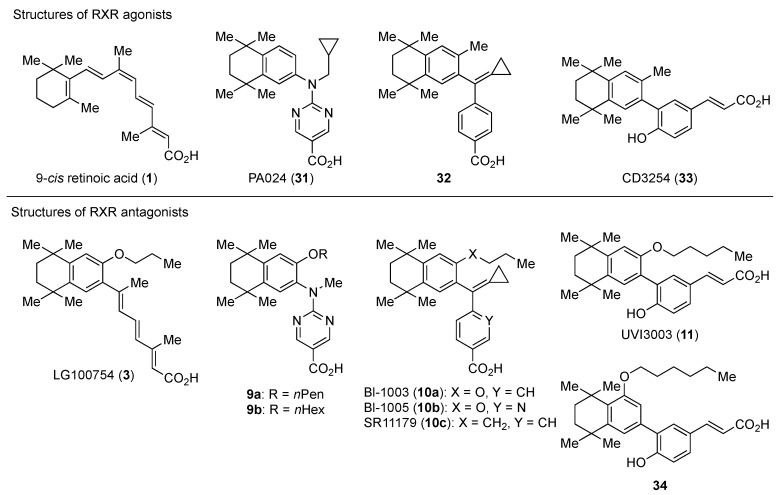
Chemical structures of RXR agonists and RXR antagonists having a long-chain alkoxy group.

The chemical structures of RXR antagonists in this category are illustrated in Table 3. LG100754 (**3**) was reported as the first RXR antagonist in 1996 [42]. Prior to that, in 1994, Boehm et al. had noted that some compounds having RXR binding affinity, but not showing RXR agonist activity, might exhibit RXR antagonistic activity [73]. Compound **3** was designed by introducing an *n*-propoxy group into the 3′-position of the backbone of tetrahydrotetramethylnaphthyl octatrienoic acid, whose chemical structure is similar to that of 9-*cis*-retinoic acid (**1**) (Figure 3). A similar compound, AGN195393 (**4**) [43], was also reported. Compound **3** showed IC_50_ = 16 nM against 32 nM **2** (EC_50_ = 33 nM) [73] in reporter assay for RXRα in CV-1 cells. Although initially identified as an RXR homodimer antagonist, subsequent experiments revealed that **3** acts as an agonist toward RAR/RXR [74], PPARα/RXR [75], and PPARγ/RXR [76,77]. Although **3** has been reported to act as a ‘phantom ligand’ activating RAR via allosteric control through the binding to RXR [74], it is revealed that the activation of RAR/RXR by **3** is caused by a direct binding of **3** to RAR that stabilizes co-activator interactions [78].

Ro26-5450 (**5**) [44] and LG101506 (**6**) [45] have a (2*E*,4*E*,6*Z*)-7-(2-alkoxy-3,5-di-alkylbenzene)-3-methylocta-2,4,6-trienoic acid scaffold. Compound **6** binds to RXRα at low concentrations and shows RXR antagonist activity, but a synergistic effect with an agonist of PPARγ was also found. Subsequently, **7**, which has a ring structure at the 6 and 7 positions of the trienoic acid structure of **6**, and **8**, which has another ring structure at the 4 and 5 positions of **7**, were created [47,48]. Compound **8** shows more potent RXR antagonist activity than **6** [47]. Their *K_i_* values for RXRα in the presence of [^3^H]9-*cis* retinoic acid are 3 nM (**6**), 9.9 nM (**7**), and 3 nM (**8**). Although the IC_50_ values toward RXRα in reporter assay using CV-1 cells were also reported as 8 nM (**6**), 10.3 nM (**7**), and 8 nM (**8**), the RXR agonist and the concentration used were not mentioned [45,46,47]. Since these RXR ligands activate specific heterodimers, the authors refer to the compounds as “selective RXR modulators” [45].

PA451 (**9a**) and PA452 (**9b**) are RXR antagonists having a pentoxy or a hexoxy group at the ortho position of the amino group on the benzene ring forming the tetramethyltetraline structure of an *N*-methyl derivative of RXR agonist PA024 (**27**). These compounds inhibit RXR/RAR heterodimers [48]. The *p*A_2_ value of **9b** in the presence of RXR agonist NEt-TMN (**36**, EC_50_ = 5.28 nM) [49] was determined as 7.11 from a Shild plot [50].

Bl-1003 (**10a**) [51] is a propoxy derivative of RXR agonist **28** [79]. Compounds **10b** and **10c** were designed by replacing the benzoic acid of **10a** with nicotinic acid and the propoxy group of **10a** with a butyl group, respectively. Reporter assay toward RXRα using 0.1 μM **1** in CV-1 cells gave IC_50_ = 1100 nM (**10a**), >10,000 nM (**10b**), and 67 nM (**10c**), respectively [52]. Interestingly, although **10c** showed a 10-times-greater *K_d_* value than **10a** in a competition test using tritium-labeled **1**, the antagonism in the reporter assay was 20 times more potent.

UVI3003 (**11**) is an RXR antagonist obtained by converting the 3′-methyl group of RXR agonist CD3254 (**33**) [54] to a pentoxy group. In this study, the authors synthesized analogs with an alkyl chain ranging from C1 to C6 in length, and evaluated RXR agonistic and antagonistic activities. Compounds having a short alkoxy side chain act as partial or weak RXR agonists, but when the number of carbons is more than 3, they show RXR antagonist activity. Among them, **11** shows potent RXR antagonistic activity. Since **34**, the positional isomer of **11**, shows only weak RXR antagonist activity, the position of the alkoxy group is important for the activity [80]. Compound **11** showed IC_50_ = 0.24 μM against 10 nM IRX4204 (formerly designated AGN194204 and NRX 194204, RXR agonist) [53] in a reporter assay for RXRα in COS-7 cells [55].

### 3.2. RXR Antagonists Possessing Another Side Group

**Figure 4:**
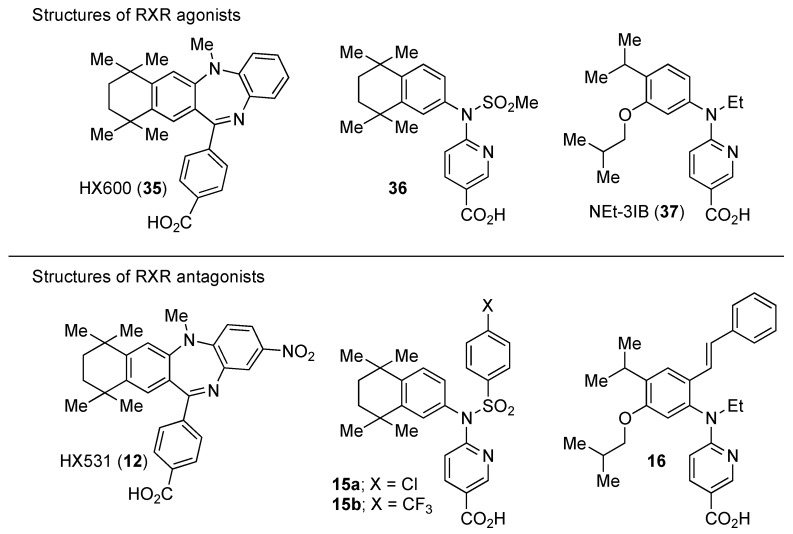
Chemical structures of RXR agonists and RXR antagonists possessing another side group instead of the alkoxy group on an RXR agonist structure.

RXR antagonists possessing another side group instead of the alkoxy chain are summarized in Figure 4 and Table 4.

HX531 (**12**) was designed by introducing a nitro group into the structure of the diazepinylbenzoic acid derivative RXR agonist HX600 (**35**) [56]. Compound **12** showed IC_50_ = 1.0 μM against 10 nM IRX4204 in a reporter assay toward RXRα in COS-7 cells [55]. Compound **12** has been reported to show antagonism towards not only RXR, but also RAR [56]. It also shows antagonistic activity against RAR/RXR or PPARγ/RXR heterodimers [7]. Compound **12** shows a hypoglycemic effect in an animal model of type 2 diabetes, and is thought to improve insulin resistance through antagonism to the PPARγ/RXR heterodimer [7]. An improvement of leptin resistance was also reported [81]. However, the *C*_max_ value at 100 mg/kg oral administration of **12** to mice was 4.1 μg/mL (8.5 μM). Two-week administration of diet containing **12** at 0.1% weight showed a hypoglycemic effect [7]. For the purpose of improving the oral availability of **12**, **13a**, and **13b** were created [57]. When they were orally administered to rats at 1 mg/kg, the *C*_max_ values were 468 nM and 519 nM, respectively. Further development of these structures yielded **13c**, which was reported to show a hypoglycemic effect in KK-Ay mouse, a type 2 diabetes model [58].

Compound **14** has a boron cluster (carborane) at the hydrophobic site instead of a tetramethyltetraline structure [59]. At 1 μM, **14** completely represses RXRα transcription induced by 10 nM RXR agonist PA024 (**31**).

Morishita and colleagues produced new RXR antagonists, **15a** and **15b**, having a sulfonamide on an amino linking group instead of the *N*-ethyl group of NEt-TMN (**36**) [29]. However, their RXR antagonist activity was weaker than that of HX531 (**12**).

To reduce the lipid solubility of existing RXR agonists, the RXR full agonist NEt-3IB (**37**, EC_50_ = 19 nM), which has an isobutoxy group at a hydrophobic site, was designed [27,82]. The para position to the isobutoxy group on the benzene ring is electron-rich because this position is also at the ortho position relative to the nitrogen atom of the amino linking group. Therefore, it is easily halogenated. A new RXR antagonist **16**, which has a stilbene structure, was created by transformation of an iodine precursor using a palladium catalyst [50]. The *p*A_2_ value of **16** toward RXRα agonist NEt-TMN (EC_50_ = 5.28 nM) [49] was 8.23 based on a Shild plot, while that of PA452 (**9b**) was 7.11; thus, **16** is one of the strongest RXR antagonists discovered thus far.

### 3.3. RXR Antagonists Discovered among Natural Products or by Docking Simulation or High-Throughput Screening

The chemical structures and assay data of RXR antagonists classified in this category are shown in Table 5.

Danthron (**17a**), a component of rhubarb, used in Chinese medicine, showed RXR antagonist activity with IC_50_ = 0.11 μM for 1 μM **1** in a reporter assay for Gal4-RXRα-LBD in HEK293T cells [60]. The *K_d_* value for RXRα is 6.2 μM. Compound **17a** shows antagonist activity toward not only RXR homodimer, but also heterodimers such as PPARγ/RXRα and LXRα/RXRα. Compound **17a** has also been evaluated in vivo and was found to improve insulin resistance in DIO mice. Rhein (**17b**), another compound derived from rhubarb, likewise shows RXR antagonist activity with IC_50_ = 0.75 μM for **1** in the same assay system [61].

β-Apo-13-carotenone (**18**), which is produced by β-carotene cleavage, antagonizes RXRα activation by **1** through receptor tetramerization, which stabilizes the inactive state [62]. Though competition assay against **1** in a reporter assay in COS-7 cells has been investigated, the IC_50_ value was not described.

*R*-Etodolac (**19**), a non-steroidal anti-inflammatory drug (NSAID), induces apoptosis of tumor cells in a mouse model of prostate cancer [63]. Zhang et al., reported that **19** acts as an antagonist of RXRα and down-regulates RXR. A competition assay with 38.1 nM [^3^H]**1** revealed that the IC_50_ value of **19** is about 200 μM. After this study, sulindac (**20**), another NSAID, was also found to bind to RXRα and induce apoptosis [64]. The IC_50_ value of **20** in competition assay for [^3^H]**1** is 82.9 μM. K-80003 (**21a**) was created to improve the affinity for RXR (IC_50_ = 2.4 μM),, and to eliminate COX inhibition [65,66]. Though K-8008 (**22b**), which has a tetrazole instead of the carboxylic acid moiety of **21a**, showed a slightly decreased affinity for RXRα (IC_50_ = 16.8 μM), crystal structure analysis showed that it binds at the RXRα interface and stabilizes the tetramer of RXR [65].

Zhang et al., also discovered triptolide (**22a**) [67], which has antagonistic activity against RXRα and induces apoptosis, as well as NSC-640358 (**23**) [69], by virtual screening. The *K_d_* value of **23** for RXRα is 15.7 μM. Furthermore, they conducted a one hybrid assay using their in-house compound library and identified **24** and **25**, which are nitrostyrene derivatives, as RXRα modulators [70]. They detected RXR agonistic activity in the mammalian one-hybrid assay using Gal4-DBD-RXRα-LBD, and antagonistic activity in reporter assay using the full-length RXR homodimer. Zhang et al., demonstrated that nitrostyrene derivatives **24** and **25** could inhibit the TNFα/NFκB signaling pathway by binding to N-terminally truncated RXRα (tRXRα), leading to TNFα and tRXRα-dependent apoptosis of cancer cells.

Moreover, Zhang et al., identified **26** and **27** as RXR antagonists by means of virtual screening using the structure of RXRα-LBD in the complex with CD3254 (**33**) and a coactivator peptide (PDB code, 3FUG) [71]. These compounds do not bind to the ligand-binding pockets, but bind at the surface of the co-regulator binding site and inhibit co-regulator binding there. Reporter assay using 0.1 μM **1** toward RXRα in MCF-7 cells yielded IC_50_ values of 2 μM for **26** and 2.45 μM for **27**.

Zhang and colleagues also found that the statin drugs fluvastatin (**28**) and pitavastatin (**29**) are RXR antagonists by virtual screening of an FDA-approved drug database [72]. Further structure optimization of **28** afforded **30**, whose *K_d_* value for RXRα is 5.1 μM, which is lower than that of danthron (**17a**).

## 4. Evaluation of RXR Antagonistic Activity

Though various RXR antagonists have been reported so far, their antagonistic activity has been evaluated in various ways, i.e., in terms of the dissociation constant (*K_i_* value) using a tritium-labeled ligand such as 9-*cis*-retinoic acid (**1**), the binding constant obtained by the SPR method, the *K_d_* value, the IC_50_ value, and *p*A_2_ against an RXR agonist in reporter assays (Table 3, Table 4 and Table 5).

The dissociation constant has been measured by using radioisotopes. However, this technique is complicated and requires special laboratory equipment, as well as disposal arrangements for radioactive waste. So far, no method using a fluorescent ligand has been established. Additionally, even if the binding ability to the receptor is detected, poor membrane permeability of the compound may influence the actual activity, as in the cases of **10a** and **10c** [52].

Antagonistic activity of LG100754 (**3**), the first reported RXR antagonist, was evaluated in terms of the IC_50_ value on transcriptional activation by **2** in reporter gene assays using CV-1 cells [42]. Similarly, PA452 (**9b**) [48] and UVI3003 (**11**) [54] were evaluated using PA024 (**31**) and CD3254 (**33**) as agonists, respectively. Since the activity differs depending on the coexisting RXR agonist, it is difficult to compare the observed potencies. The most widely used RXR agonist for reporter gene assays is **1** at the concentration of 0.1 μM. Therefore, it may be better to use this method as one index of activity in screening for new RXR antagonists.

The *p*A_2_ value is used as an index of competitive antagonist activity. It is the negative logarithm of the molar concentration of the competitive antagonist required to shift the agonist’s EC_50_ to two-fold higher concentration. The *p*A_2_ value is also consistent with the affinity constant for the receptor [83]. Thus, it is desirable to include this method in a more rigorous evaluation of antagonist activity. However, in order to obtain these data, it is necessary to obtain a capacity activity curve of the agonist at three different antagonist concentrations at minimum. Compounds **9b** and **16** have been evaluated using the *p*A_2_ value as an indicator of competitive antagonist activity [50].

RXR forms not only RXR homodimers, but also heterodimers with various nuclear receptors [2]. Therefore, it is interesting to know whether RXR antagonists act as homodimer antagonists and/or heterodimer antagonists. Though **3** was found as an RXR homodimer antagonist, subsequent experiments revealed that it also acts as an agonist toward RAR/RXR [74], PPARα/RXR [75], and PPARγ/RXR [76,77]. Compound **6** has been found to show a synergistic effect in the presence of an agonist of PPARγ [45]. Compound **9b** selectively antagonizes RXR in RXR/RAR heterodimer [48]. One micromole of **12** suppressed the activity of 100 nM rosiglitazone (PPARγ agonist) toward PPARγ/RXR to about a half [7]. Compound **17a** has antagonistic activity not only towards the RXR homodimer, but also towards heterodimers such as PPARγ/RXRα, FXR/RXRα, LXRα/RXRα, etc. [60]. However, there was no description of the concentration of each agonist for partner receptors. Among them, for LXR/RXR, T0901317 [84] with an EC_50_ of 20 nM for LXRα was used at 5 μM. Based on these facts, it seems necessary to standardize assay systems for heterodimers.

## 5. Latest Research on RXR Antagonists

Here, we will briefly summarize research on RXR antagonists reported in the last five years, and then consider the prospects for RXR antagonists.

LG100754 (**3**) was reported to have a protective effect against oxidative stress in retinal pigment epithelial cells [85]. This effect is thought to be caused by activation of PPARγ/RXR.

PA452 (**9b**) was reported to decrease an infection marker concentration-dependently in an HBV infection model using human hepatic stem cells [86]. It is considered that **9b** suppresses transcription of viral RNA in HBV-infected hepatocyte-like cells by antagonizing RXR.

Teratogenicity of UVI3003 (**11**) was studied using zebrafish and *Xenopus* [87,88]. A difference in gene expression in *Xenopus* eggs was found depending on the exposure time to **11** [89]. In 2017, **11** was found to activate PPARγ in a reporter assay using *Xenopus* embryos. Moreover, studies using *Xenopus* treated with RXR agonist bexarotene (**2**) or **11** revealed that T3-dependent gene expression was altered during transformation of tadpoles [90].

Ro26-5405 (**5**) is reported to block T helper 2 differentiation and to prevent allergic lung inflammation [8]. The mechanism was suggested to be inhibition of Th2 differentiation by antagonizing RXR. In addition, in an atopic dermatitis model mouse, **11** was used as a tool to investigate the expression of thymic stromal lymphopoietin (TSLP), which is triggered in atopic dermatitis and is involved in suppression [91]. TSLP is an IL-7-like cytokine and was shown to be a master switch of allergic inflammation at the epithelial cell–dendritic cell interface, leading to allergic sensitization. It is reported that the expression of TSLP involves RARγ/RXR.

Huang et al. used **12** as a tool to show that activation of RXR has a protective effect against hypoxia-reoxygenation disorder in H9c2 cardiomyocytes [92]. Franklin and colleagues revealed that phagocytosis and remyelination of myelin debris accompanying aging progressed upon activation of RXR using **12** [93]. Kajta et al. reported that apoptotic neurotoxic activity of 4-para-nonylphenol occurs simultaneously with RXR activation and a decrease in classical estrogen receptor signaling. They found that the effect of 4-para-nonylphenol on mitochondrial membrane potential was canceled by **12**, indicating that this neurotoxicity involves activation of RXR [94]. Compound **12** is also reported to decrease both mobility and growth of *Trichuris muris* (a parasite) in vitro, indicating its potential as an anthelmintic drug [95]. RXR is negatively regulated by **1** and **12** through a nongenomic effect on platelets and thrombus formation [96].

Compound **12** is also used as a tool to investigate the influence of environmental hormones on RXR. For example, the mechanism of neurotoxicity by dichlorodiphenyldichloroethylene (DDE) [97], the effect of tributyltin on osteogenesis [98], and the toxicity of organotin [99] were found to involve transcriptional activation of RXR.

Zhang and colleagues found that *R*-etodolac (**19**), a NSAID, induces an antitumor effect via antagonistic activity toward RXRα, and also induces degradation of RXRα via the ubiquitin-proteasome system [63]. Subsequently, they also found RXR antagonist activity of sulindac (**20**), another NSAID. They suggested that nongenomic action of an N-terminally truncated RXRα (tRXRα) could play a role in the crosstalk with TNFα signaling in cancer cells [64,100]. tRXRα, which is produced by proteolytic cleavage of full-length RXRα, is highly expressed in a variety of tumor cells and tissues [101,102]. Furthermore, **20** was structurally developed to afford compounds **21a** and **21b** [64,65]. Crystal structure analysis of **21b** in RXRα revealed that it binds to the RXR interface rather than the ligand-binding pocket, stabilizing RXR tetramers [65].

Similarly, Zhang et al., discovered triptolide (**22a**) in a natural product library [67]. Compound **22a** regulates the survival of tRXRα-dependent cancer cells by apoptosis induction. Furthermore, **22a** was structurally converted to TRC4 (**22b**), and **22b** showed tRXRα-selective antagonism without transcriptional activation of RXRα [68]. In addition, NSC-640358 (**23**), which was discovered by virtual screening (*K_d_* = 15.7 μM), induces apoptosis of cancer cells [69]. Compound **23** has been reported to inhibit the transcriptional activation of RXR homodimer by **1**, but the IC_50_ value was not given.

In addition, Zhang et al., carried out one-hybrid assay with a compound library and found nitrostyrene derivatives **24** and **25** as RXR modulators [70]. Although these compounds showed RXR activity in mammalian one-hybrid assay using Gal4-DBD-RXRα-LBD, they showed antagonist activity in reporter assays using full-length RXR homodimer. Interestingly, **24** and **25** stabilize the RXR homodimer, unlike **21b**. Size-exclusion chromatography indicated that the structure of the homodimer differs from the activated structure. These compounds have no activity to down-regulate tRXRα. Compounds **26**, **27** were also discovered by virtual screening [71].

## 6. Important Points in the Use of RXR Antagonists

Some RXR antagonists reported to date show agonistic activity on RXR heterodimers. For example, LG100754 (**3**), in addition to antagonism of the RXR homodimer [43], shows agonist activity toward RAR/RXR [74], PPARα/RXR [75], and PPARγ/RXR. [76,77] UVI3003 (**11**) also shows agonistic activity for PPARγ/RXR [55]. HX531 (**12**), the most widely used RXR antagonist in vivo, has also been reported to antagonize RAR. [7] Chen et al. reported that down-regulation of RXRα leads to cyclooxygenase-2 (COX-2) expression and prostaglandin E2 (PGE2) production in aged macrophages [103]. These data were obtained by administering **12** to mice. However, **12** was administered at a high concentration of 10 mg/kg i.p., every 24 h for seven days. The C_max_ of **12** in mice after 100 mg/kg oral administration was only 4.1 µg/mL (8.5 μM) [7]. In order to improve oral absorption, **13a**, **13b** and **13c** were created [57,58]. However, although **13a** and **13b** give C_max_ values of approximately 500 nM after oral administration to rats at 1 mg/kg, there is no report as yet on their activities toward RXR heterodimers.

## 7. Conclusions

RXR antagonists are of increasing interest because of their therapeutic effects, i.e., hypoglycemic effect in type 2 diabetes models and anti-tumor effect via tRXRα. However, currently available RXR antagonists require high dosages in vivo when orally administered because of their poor absorption, and some of them activate heterodimers. Thus, there is still a need to develop new RXR antagonists to overcome these problems, and such compounds would be promising drug candidates, as well as useful experimental tools for biological studies on the roles of nuclear receptors.

## Figures and Tables

**Table 1:** Definitions of terms and abbreviations concerning nuclear receptors (adapted and modified from [3]).

Term	Definition/Description/Examples
AF-1	Activation function-1. AF-1 consists of the N-terminal region (domains A/B), which can operate autonomously. This region can interact with cofactors such as co-activators or other transcription factors. The activation is independent of ligand binding. The activity of AF-1 is regulated by growth factors acting through the MAP kinase pathway.
AF-2	Activation function-2. AF-2 is the C-terminal helix 12, located in domain E, which mediates ligand-dependent transactivation.
DBD	DNA-binding domain. Domain C of nuclear receptors. This region binds to a specific DNA sequence, called the hormone response element (HRE).
LBD	Ligand-binding domain. Domain E of nuclear receptors. The LBD contains (1) a dimerization surface, which mediates interaction with partner LBDs; (2) the LBP; (3) a co-regulator binding surface, and 4) an activation function helix, termed AF-2.
LBP	Ligand-binding pocket (LBP), which interacts with small molecules. The LBP is generally located behind helix 3 and in front of helices 7 and 10, and is lined with mostly hydrophobic amino acids.
Ligands for NRs	Compounds that bind reversibly to NRs at the C-terminal LBP.
Agonists	Ligands that induce an active conformation of the receptor
Inverse agonists	Ligands that can promote co-repressor recruitment.
Antagonists	Ligands that produce a conformation and an action of the receptor distinct from that produced by an agonist.
Partial agonists	Agonists that in a given tissue, under specific conditions, cannot elicit as large an effect (even when applied at high concentration, so that all the receptors should be occupied), as can another agonist acting through the same receptors in the same tissue
NR modulators	Compounds that bind to NRs, which include ligands, SNuRMs, and SNuRDs [26].
Orthosteric modulators	Compounds that bind to the same site of endogenous ligands.
Allosteric modulators	Compounds that bind to the different site of endogenous ligands. The term “allo-” means “other”.
Positive allosteric modulators (PAMs)	Allosteric modulators that induce an amplification of the effect of the primary ligand.
Negative allosteric modulators (NAMs)	Allosteric modulators that reduce the effect of the primary ligand.
Silent allosteric modulators (SAMs)	Allosteric modulators that occupy the allosteric binding site and behave functionally neutral; also called neutral or null modulators.
SNuRMs	Selective nuclear receptor modulators. Selective ligands with partial function-, cell-, and/or promoter-specific action.
SNuRDs	Selective nuclear receptor Down-regulators. Compounds that cause NR to be degraded and thus down-regulated. A subclass of antagonists. Fluvestrant is a selective estrogen receptor down-regulator (SERD) [31].
Selective agonists and antagonists	Ligands with an affinity difference (preferably greater than 100-fold) between their primary target and other receptors.

**Table 2:** Classification and nomenclature of nuclear receptors (adapted and modified from [32]).

Name	Subtypes	Nomenclature	Sequence	References
TR	α	NR1A1	DR4	[33]
	β	NR1A2		[33]
RAR	α	NR1B1	DR2, DR5	[33,34,35]
	β	NR1B2		[33]
	γ	NR1B3		[33,36]
PPAR	α	NR1C1	DR1	[33]
	β/δ	NR1C2		[33,34,35]
	γ	NR1C3		[33,34,35,36]
LXR	α	NR1H1	DR4	[33,34,35,36]
	β	NR1H2		[33,34,35,36]
VDR		NR1I1	DR3	[33,34,35,36]
PXR		NR1I2	DR3–5	[37]
LXR	α	NR1H1	DR4	[33,34,35,36]
FXR		NR1H4	IR1 *	[38]
RXR	α	NR2B1	DR1	[33,34,35,36]
	β	NR2B2		[33,34,35,36]
	γ	NR2B3		[33,34,35,36]
Nur77		NR4A1	DR5	[36,39,40,41]
Nurr1		NR4A2	DR5	[36,40]

* IR Inverted repeat.

**Table 3:** Chemical structures, binding affinities, and RXR antagonistic activities of RXR antagonists having an alkoxy side chain on an RXR agonistic scaffold.

Compounds	Structures	Binding	Transactivity (RXRα)	Ref.
LG100754 (**3**)	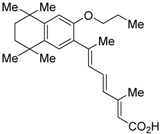	*K_i_* = 3 nM (RXRα, [^3^H]**1**) *K_i_* = 8 nM (RXRα, [^3^H]**2**)	IC_50_ = 16 nM (vs. 32 nM **2**, CV-1 cells)	[42]
AGN195393 (**4**)	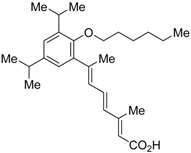	N.D.	N.D.	[43]
Ro26-5405 (**5**)	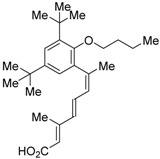	*K_i_* = 0.9 nM (RXRα, [^3^H]**2**)	N.D.	[43,44]
LG101506 (**6**)	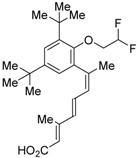	*K_i_* = 3 nM (RXRα, [^3^H]**1**) *K_i_* = 3 nM (RXRα, [^3^H]**2**)	IC_50_ = 8 nM (CV-1 cells)	[43,45]
**7**	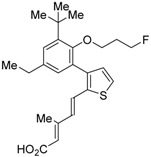	*K_i_* = 9.9 nM (RXRα, [^3^H]**1**)	IC_50_ = 10.3 nM (CV-1 cells)	[46]
**8**	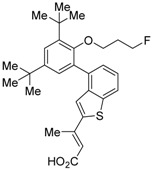	*K_i_* = 3 nM (RXRα, [^3^H]**1**)	IC_50_ = 8 nM (CV-1 cells)	[47]
PA451 (**9a**) R = *n*-Pen	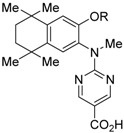	N.D.	N.D.	[48]
PA452 (**9b**) R = *n*-Hex		N.D.	*p*A_2_ = 7.11 (vs. NEt-TMN: EC_50_ = 5.28 nM [49], COS-1 cell)	[48,50]
Bl-1003 (**10a**) X = O, Y = CH	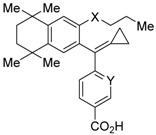	*K_d_* = 26 nM (RXRα-LBD, fluorescence titration) IC_50_ = 46 nM (RXRα-LBD, [^3^H]**1**)	IC_50_ = 1100 nM (vs. **1** @ 0.1 μM, CV-1 cells)	[51,52]
Bl-1005 (**10b**) X = O, Y = N		*K_d_* = 329 nM (RXRα-LBD, fluorescence titration) IC_50_ = 1200 nM (RXRα-LBD, [^3^H]**1**)	IC_50_ ≥ 10,000 nM (vs. **1** @ 0.1 μM, CV-1 cells)	[51,52]
SR11179 (**10c**) C = CH_2_, Y = CH		*K_d_* = 15 nM (RXRα-LBD, fluorescence titration) IC_50_ = 450 nM (RXRα-LBD, [^3^H]**1**)	IC_50_ = 67 nM (vs. **1** @ 0.1 μM, CV-1 cells)	[51,52]
UVI3003 (**11**)	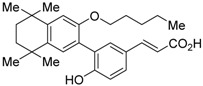	N.D.	IC_50_ = 0.24 μM (vs. IRX4204: EC_50_ = 0.2 nM [53] @ 10 nM, COS-7 cells)	[54,55]

N.D. means that the datum was not described in the cited manuscript.

**Table 4:** Chemical structures, binding affinities, and RXR antagonistic activities of RXR antagonists having a non-alkoxy side chain or another structure on an RXR agonistic scaffold.

Compounds	Structures	Binding	Transactivity (RXRα)	Ref.
HX531 (**12**)	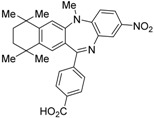	N.D.	IC_50_ = 1.0 μM (vs. IRX4204: EC_50_ = 0.2 nM [53] @ 10 nM, COS-7 cells)	[55,56]
**13a** R^1^ = Et, R^2^ = NHSO_2_-(3-CF_3_)Ph, X = H	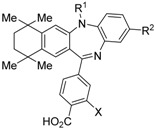	N.D.	IC_50_ = 0.095 μM (vs. **1** @ 20 nM, HEK-293 cells)	[57]
**13b** R^1^ = *n*-Pr, R^2^ = NHSO_2_-(3-CF_3_)Ph, X = H		N.D.	IC_50_ = 0.076 μM (vs. **1** @ 20 nM, HEK-293 cells)	[57]
**13c** R^1^ = Et, R^2^ = CN, X = F		N.D.	IC_50_ = 0.50 μM (vs. **1**, HEK-293 cells)	[58]
**14**	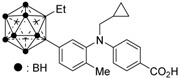	N.D.	N.D.	[59]
**15a** X = Cl	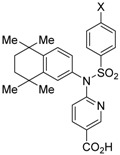	N.D.	IC_50_ = 4.1 μM (vs. **2** @ 10 nM, COS-1 cells)	[29]
**15b** X = CF_3_		N.D.	IC_50_ = 3.2 μM (vs. **2** @ 10 nM, COS-1 cells)	[29]
**16**	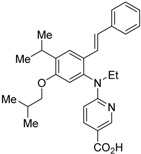	N.D.	*p*A_2_ = 8.23 (vs. NEt-TMN: EC_50_ = 5.28 nM [49], COS-1 cells)	[50]

N.D. means that the datum was not described in the cited manuscript.

**Table 5:** Chemical structures, binding affinities, and RXR antagonistic activities of RXR antagonists from natural products or others.

Compounds	Structures	Binding	Transactivity (RXRα)	Ref.
Danthron (**17a**) R = H	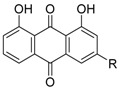	*K_d_* = 6.2 μM (RXRα-LBD, SPR) *K_d_* = 7.5 μM (RXRα-LBD, ITC)	IC_50_ = 0.11 μM (vs. **1** @ 0.1 μM, HEK-293T cells)	[60]
Rhein (**17b**) R = CO_2_H		N.D. *	IC_50_ = 0.75 μM (vs. **1** @ 0.1 μM, HEK-293T cells)	[61]
β-Apo-13-carotenone (**18**)	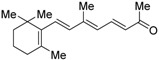	N.D.	IC_50_ value is not described (vs. **1** @ 0.01~1000 nM, COS-7 cells)	[62]
*R*-Etodolac (**19**)	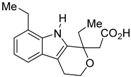	IC_50_ ≈ 200 μM (RXRα-LBD, [^3^H]**1**)	N.D.	[63]
Sulindac sulfide (**20**)	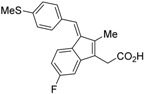	IC_50_ = 80 μM (RXRα-LBD, [^3^H]**1**)	N.D.	[64]
K-80003 (**21a**) X = F, R = CO_2_H	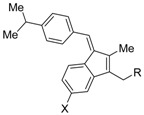	IC_50_ = 2.4 μM (RXRα-LBD, [^3^H]**1**)	N.D.	[64,65]
K-8008 (**21b**) X = H R = 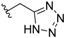		IC_50_ = 16.8 μM TR-FRET, GST-RXRα-LBD, **1** @ 10 nM)	IC_50_ = 13.2 μM (vs. **1** @ 100 nM, HCT-116 cells)	[65,66]
Triptolide (**22a**) R = H	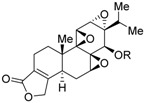	N.D.	N.D.	[67]
TRC4 (**22b**) R = 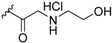		N.D.	N.D.	[68]
NSC-640358 (**23**)	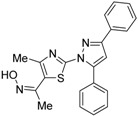	*K_i_* = 15.7 μM (RXRα-LBD, [^3^H]**1**)	N.D.	[69]
**24**	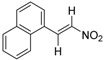	*K_i_* = 0.28 μM (RXRα-LBD, [^3^H]**1**)	N.D.	[70]
**25**	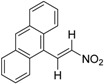	*K_i_* = 0.81 μM (RXRα-LBD, [^3^H]**1**)	N.D.	[70]
**26**	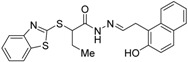	N.D.	IC_50_ = 2 μM (vs. **1** @ 0.1 μM, HEK-293T cells)	[71]
**27**	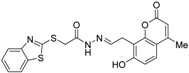	K_d_ = 488 nM (RXRα-LBD, SPR)	IC_50_ = 2.45 μM (vs. **1** @ 0.1 μM, HEK-293T cells)	[71]
Fluvastatin (**28**)	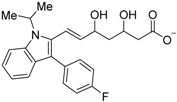	*K_d_* = 11.04 μM (RXRα-LBD, SPR)	IC_50_ value is not described. (vs. **1** @ 100 nM, MCF-7 cells)	[72]
Pitavastatin (**29**)	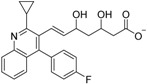	*K_d_* = 13.30 μM (RXRα-LBD, SPR)	IC_50_ value is not described. (vs. **1** @ 10 nM, MCF-7 cells)	[72]
**30**	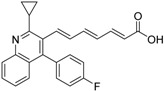	K_d_ = 5.12 μM (RXRα-LBD, SPR)	IC_50_ value is not described. (vs. **1** @ 100 nM, MCF-7 cells)	[72]

N.D. means that the datum was not described in the cited manuscript.

## Abbreviations

i.p.	Intraperitoneal injection
NFκB	Nuclear factor-kappa B
NR	Nuclear receptor
Th2	T helper type 2
TNFα	Tumor necrosis factor alpha
TR	Thyroid hormone receptor
VDR	Vitamin D receptor
PXR	Pregnane X receptor
